# Blind quality assessment of multi-exposure fused images considering the detail, structure and color characteristics

**DOI:** 10.1371/journal.pone.0283096

**Published:** 2023-04-06

**Authors:** Lijun Li, Caiming Zhong, Zhouyan He

**Affiliations:** 1 School of Automation, Southeast University, Nanjing, China; 2 College of Science and Technology, Ningbo University, Ningbo, China; 3 School of Automation, Qingdao University, Qingdao, China; University of Engineering & Technology, Taxila, PAKISTAN

## Abstract

In the process of multi-exposure image fusion (MEF), the appearance of various distortions will inevitably cause the deterioration of visual quality. It is essential to predict the visual quality of MEF images. In this work, a novel blind image quality assessment (IQA) method is proposed for MEF images considering the detail, structure, and color characteristics. Specifically, to better perceive the detail and structure distortion, based on the joint bilateral filtering, the MEF image is decomposed into two layers (i.e., the energy layer and the structure layer). Obviously, this is a symmetric process that the two decomposition results can independently and almost completely describe the information of MEF images. As the former layer contains rich intensity information and the latter captures some image structures, some energy-related and structure-related features are extracted from these two layers to perceive the detail and structure distortion phenomena. Besides, some color-related features are also obtained to present the color degradation which are combined with the above energy-related and structure-related features for quality regression. Experimental results on the public MEF image database demonstrate that the proposed method achieves higher performance than the state-of-the-art quality assessment ones.

## Introduction

Multi-exposure fusion (MEF) images are obtained from a series of images with different exposure levels, which can offer richer information than any of the input images [1]. Compared with high dynamic range (HDR) images, the MEF image bypasses the process of tone mapping for displaying on common devices, and it can also obtain similar imaging effects to the HDR images. However, nothing is perfect, the existing MEF methods cannot achieve satisfactory effects for all MEF image sequences [2, 3]. Always, the MEF image suffers from detail loss, structure destruction and color degradation which affect its visual quality. Therefore, it is necessary to develop some MEF image quality assessment (IQA) methods for comparing the performance of different MEF methods.

According to the utilization rate of the reference information, the IQA methods can be categorized into three types [4], that are full-reference (FR) methods, reduced-reference (RR) methods, and no-reference (NR) methods. FR and RR methods need reference information for quality prediction, and NR methods do not require any reference information. However, due to the special way of MEF image acquisition, there are multiple reference images that cannot be directly compared with the fused images. Hence, NR methods are suitable for the MEF images.

In this paper, considering the detail, structure, and color distortion of MEF images, an NR-IQA method for MEF images is proposed based on joint bilateral filtering. Specifically, the MEF image is decomposed into the energy layer and structure layer by the joint bilateral filter, and then the two decomposed results are utilized to extract some quality-sensitive features for the distortion description. Meanwhile, color-related features are also extracted to perceive color distortion. As a whole, this paper presents the following contributions.

1. Inspired by the image fusion strategy, joint bilateral filtering is utilized for MEF image decomposition to obtain the energy layer and structure layer, which can better facilitate the extraction of distortion features.
2. Considering the detail and structure distortion of MEF images, energy-related and structure-related features are extracted from the two decomposition layers, respectively, which are relative to human visual perception.
3. In terms of color distortion introduced, some color-related features are also extracted. To obtain better quality prediction performance, feature selection is conducted based on random forest. Experimental results show that the proposed method is better than other competing methods.

The remainder of this paper is arranged as follows. Section 2 presents the related work about the IQA methods. The details of the proposed method are described in Section 3. Experimental results and analyses are given in Section 4. Section 5 draws the conclusion.

## Related works

In this section, the existing multi-exposure image fusion methods and image quality assessment methods are presented here.

### Multi-exposure image fusion methods

Since the MEF image can inherit the advantages of multiple images with different exposure degrees, the MEF method has received a lot of attention. Burt et al [5] utilized the Laplacian pyramid decomposition and the pyramid decomposition for binocular and MEF images, respectively. Goshtasby et al [6] partitioned the multiple images into uniform blocks and chose the maximum information of each block for fusion. Mertens et al [7] calculate the weighting maps to blend multiple exposures via color saturation and contrast. Raman et al [8] designed a fusion method based on the bilateral filter to obtain the MEF image. Li et al [9] introduced a new quadratic optimization-based method for fusion, the great contribution is that the fine details are extracted from a vector field. Gu et al [10] fused the multi-exposure images in the gradient field, since the human visual system (HVS) is sensitive to contrasts that can be represented by local gradients. To propose fast and effective methods, Li et al [11, 12] carried out two pieces of research. The first one is a two-step work including the weight map calculation and the fused image construction. The second one decomposed the image into a base layer and a detail layer to make full use of spatial information. Ma et al [13] utilized a gradient ascent-based algorithm to implement the iterative optimization for MEF. Meanwhile, to obtain better results, Ma et al [14] designed a method based on deep learning.

### Image quality assessment methods

As mentioned in the introduction, the IQA methods include FR, RR, and NR forms [15]. The NR ones are more practical in real applications. With regard to the NR quality assessment for ordinary images, many methods rely on natural scene statistics (NSS). For example, Moorthy et al. [16] utilized NSS to design the method named distortion identification-based image verity and integrity evaluation (DIIVINE). Saad et al. [17] processed the image in the discrete cosine transform domain and established an NSS model, which is named BLINDS-II. Mittal et al. [18] proposed BRISQUE method based on the features extracted from the NSS distribution. Liu et al. [19] extracted features from curvelet transform domain, named CurveletQA. Fang et al. [20] considered the natural statistical characteristics of images with contrast distorted must be different from those of normal images and designed a method called ContrastQA. In image processing, the gradient is very important for images, which can represent the structure information of images. In view of this, to sense structure distortion, Xue et al. [21] extracted the features combining the Laplacian of Gaussian response and gradient magnitude map, named GradLog. Li et al. [22] describe the structure distortion by the gradient-weighted histogram of local binary pattern (GWH-GLBP). Liu et al. [23] considered the role of oriented gradient (OG) in quality prediction. Besides, Gu et al. [24] proposed the NIQMC method considering the contrast distortion of the image from the local and global perspectives. Jiang et al. [25] obtained a set of features, i.e., histogram, entropy, and structure, to achieve the goal of quality prediction, which also characterized the contrast. Oszust [26] presented a feature description form by image derivatives of different orders.

Above all are designed for ordinary images, and there is still a lack of NR-IQA methods for MEF images. For the FR methods of MEF images, Ma et al. [27] measured structure consistency and luminance consistency based on structural similarity index measure (SSIM). Xing et al. [28] built a multi-scale contrast-based model inspired by the fact that the human visual system (HVS) is highly sensitive to contrast. Martinez et al. [29] implemented two stages including multi-scale computation and structural similarity score. Xu et al. [30] generated the local and global intermediate references from the input multiple images to extract features, reflecting the visual quality of fused images. In real application, the visual effect of MEF images is similar to tone-mapped images. For tone-mapped images, there exist some NR-IQA methods. For instance, Gu et al. [31] developed a tone-mapped quality index (BTMQI) taking the information, naturalness and structure into consideration. Inspired by the NSS model, Kundu et al. [32] measured the differential NSS and proposed the high dynamic range image gradient-based evaluator (HIGRADE). Yue et al. [33] simulated the color information process in the human brain to propose a novel quality assessment method, meanwhile, they further presented another method [34] in terms of color, naturalness, and structure. Besides, Jiang et al. [35] pointed out the viewing habit of humans and evaluated the quality of tone-mapped images from a local and global perspective. Wang et al. [36] also considered the local degradation characteristics and global statistical properties to present a method. Although there are a few NR-IQA methods for ordinary and tone-mapped images, it is still necessary to propose NR-IQA methods specifically for MEF images.

## The proposed method

**Fig 1:**
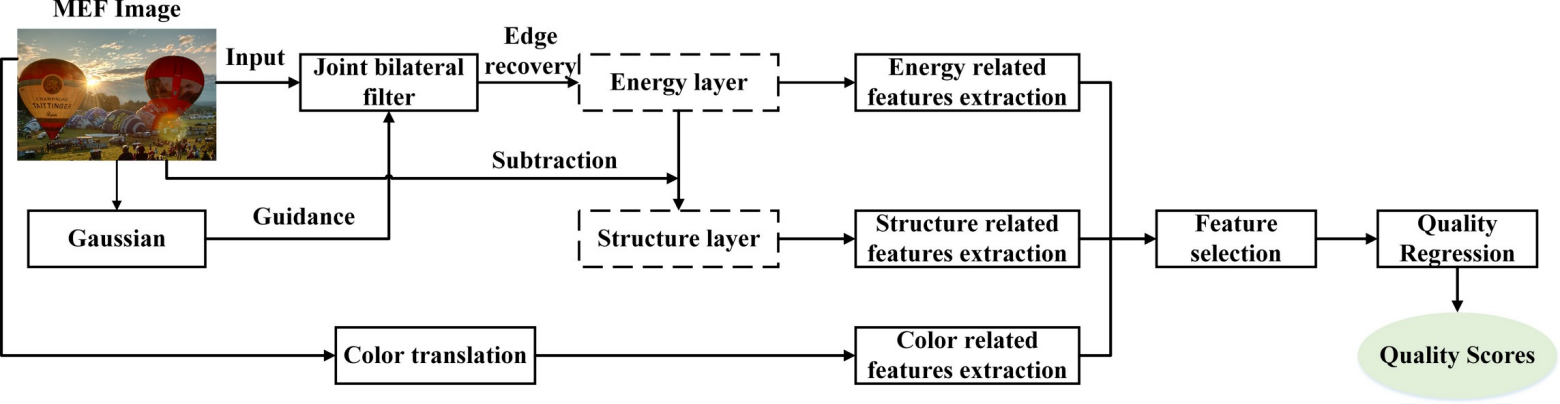
The framework of the proposed method.

In this section, the proposed method is presented in detail, and the framework is shown in Fig 1. Specifically, the joint bilateral filter is utilized to decompose the MEF image, and the obtained energy and structure layers are used to portray the detail and structure distortion of the MEF image, respectively. In addition, color-related features are also extracted to measure the colorfulness degradation. Finally, the contributions of all features are measured through the random forest to maximize the quality prediction performance.

### Image decomposition

It is well known that the averaging techniques including average filter and lowpass filter are widely used to decompose the image. However, these schemes may introduce detail loss more or less, which will damage the MEF image twice. To obtain the complementary information among the MEF image, a two-scale decomposition scheme is used based on the joint bilateral filter. As the intensity information and structure are important for the well-preservation MEF image, it is decomposed into two layers (i.e., the energy layer and structure layer). The process includes global blur and edge recovery.

For global blur, the important purpose is to disseminate the detailed information of the MEF image to the decomposed structural layer to the maximum extent. Given a MEF image ***I***, it can be processed by Gaussian filtering to achieve the blur effect, which is denoted as ***F***_σ_, defined as

$$F_{\sigma }=G_{\sigma }*I$$ (1)

$$G_{\sigma }\left(x,y\right)=\frac{1}{2\pi \sigma^{2}}\exp\left(-\frac{x^{2}+y^{2}}{2\sigma^{2}}\right)$$ (2)

where *G*_*σ*_(*x*,*y*) is Gaussian filter, and the variance is *σ*^2^.

Then, to generate the global blurred MEF image ***G***, a weighted average Gaussian filter is utilized, which can be represented as

$$G\left(m\right)=\frac{1}{N_{m}}\sum_{n\in S(m)}\exp\left(-\frac{∥m-n{∥}^{2}}{2\sigma^{2}}\right)I(n)$$ (3)

$$N_{m}=\sum_{n\in S(m)}\exp\left(-\frac{∥m-n{∥}^{2}}{2\sigma_{s}^{2}}\right)$$ (4)

where *m* and *n* denote the index of pixel coordinates, *S*(*m*) is a set of neighboring pixels of *n*, *σ*_*s*_ is the standard deviation, and *N*_*m*_ is the normalization operation.

Subsequently, the joint bilateral filter is introduced to recover the large structure of the MEF image which is destroyed by the above smooth operation, so as to obtain the energy layer, denoted as ***I***_**E**_. The process is defined as

$$I_{\text{E}}(m)=\frac{1}{N_{m}}\sum_{n\in S(m)}g_{d}\left(n-m\right)g_{r}\left(n-m\right)I\left(n\right)$$ (5)

$$N_{m}=\sum_{n\in S(m)}\exp\left(-\frac{∥m-n{∥}^{2}}{2\sigma_{s}^{2}}-\frac{∥G(m)-G(n){∥}^{2}}{2\sigma_{r}^{2}}\right)$$ (6)

where

$$g_{d}\left(n-m\right)=\exp\left(-\frac{∥m-n{∥}^{2}}{2\sigma_{s}^{2}}\right)$$ (7)

$$g_{r}\left(n-m\right)=\exp\left(-\frac{∥G(m)-G(n){∥}^{2}}{2\sigma_{r}^{2}}\right)$$ (8)

where *g*_*d*_ and *g*_*r*_ are the spatial distance function and intensity range function, respectively. They are used to set weights based on the distance among pixels and intensity differences, respectively. *σ*_*s*_ and *σ*_*r*_ control the spatial and distance weights of the bilateral filter [37], respectively.

For a MEF image ***I***, let its structure layer be ***I***_S_, which can be calculated as

$$I_{\text{S}}(x,y)=I(x,y)-I_{\text{E}}(x,y)$$ (9)

**Fig 2:**
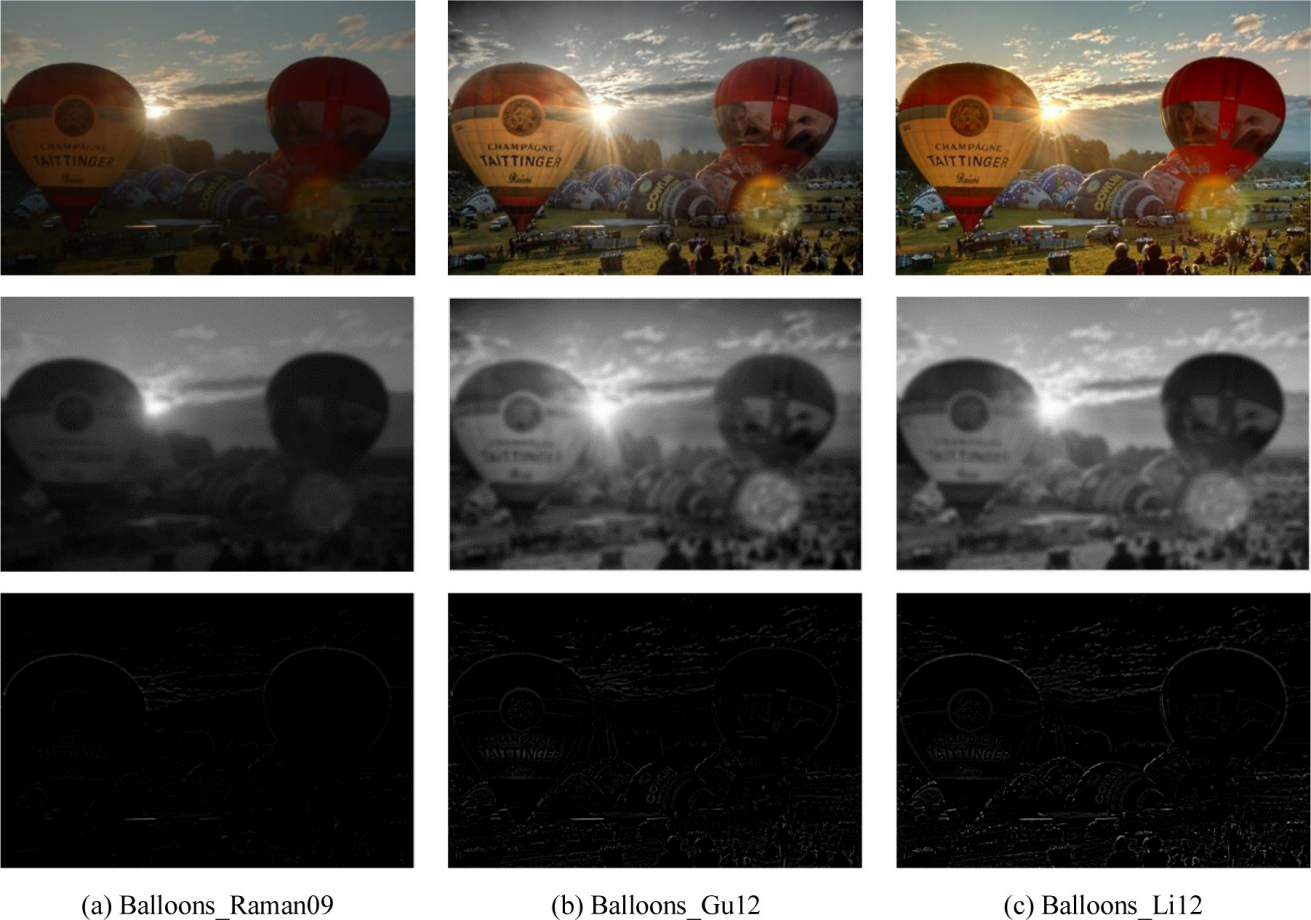
The examples of energy and structure layers obtained from the MEF images. The first line represents the MEF images which are generated by three MEF algorithms, i.e., Raman09 [8], Gu12 [10], Li12 [9]. The second line shows the corresponding energy layers of the MEF images in the first line. The third line shows the corresponding structure layers of the MEF images in the first line. (a) Balloons_Raman09, MOS = 3.17; (b) Balloons_Gu12, MOS = 5.87; (c) Balloons_Li12, MOS = 8.74.

Fig 2 shows examples of the energy and structure layers decomposed from the MEF images with different mean opinion score (MOS) values. It can be observed that Fig 2(A) shows the dim visual effect with the lowest MOS value, it loses some detailed information. This is also reflected in its corresponding energy and structure layers. The visual quality of Fig 2(B) is at a normal level, which preserves well details and structural information, and its MOS value is at a moderate level. For Fig 2(C), its detailed and structural information seems similar to Fig 2(B), but obviously, the color information is more abundant than Fig 2(B), which determines that it has the highest MOS value. The phenomenon of similar information between Fig 2(B) and 2(C) is reflected in the corresponding energy layers. However, small detail and structure differences between them can be reflected in the corresponding structure layers. From the above observation, it can be drawn that the detail, structure, and color information are important for a well-quality MEF image. Therefore, the energy-related, structure-related and color-related features will be extracted to describe the distortion of the MEF image.

### Energy-related features

The energy layer ***I***_E_ contains the details of the MEF image. Through the decomposition process, ***I***_E_ has a slightly ambiguous effect. Different energy layers will have different blurring representations. We calculated the contrast values from the edge blocks of ***I***_E_.

First, ***I***_E_ is divided into *k*×*k* blocks, *k* is set as 64, which is suggested in [38]. To judge whether the block is the edge block or the flat block, the Sobel operator is used for edge detection by calculating the proportion of edge pixels in the whole block. If greater than 0.2%, the block is the edge one. Then, let *F*_*c*_ be the feature of contrast, which can be defined as

$$F_{c}=\sum_{t=1}^{T}\sqrt{\frac{1}{k\times k}\sum_{i=0}^{k-1}\sum_{j=0}^{k-1}{\left(I_{\text{E}}{}^{\prime }\left(i,j\right)-{\bar{I}}_{\text{E}}\right)}^{2}}$$ (10)

where *I*_E_^’^(*i*, *j*) is the *i*-th, *j*-th element of the edge block with the size of *k*×*k*. *T* is the number of edge blocks in ***I***_E_, and ${\bar{I}}_{\text{E}}$ is the average intensity of all pixel values in the whole ***I***_E_.

In addition, let *F*_*e*_ be the feature of energy to represent the amount of detailed information, which can be calculated as

$$F_{e}=\frac{1}{\sum \sum I_{\text{E}}\left(i,j\right)}\sum \sum \left(I_{\text{E}}\left(i,j\right)\;*\;I_{\text{E}}\;\left(i,j\right)\right)$$ (11)

where ***I***_E_(*i*, *j*) is the pixel value of ***I***_E_ in the position of (*i*, *j*).

From the viewpoint of entropy, it can also discriminate the detailed information loss. Therefore, the entropy value of ***I***_E_ is calculated to assist the features *F*_*c*_ and *F*_*e*_. Let *F*_*t*_ be the feature of entropy, and the calculation formula is

$$F_{t}=-\sum_{l}p(l)\log_{2}\;p(l)$$ (12)

where *l* is the pixel value of ***I***_E_, and *p*(*l*) is the probability density of *l*.

Through the above calculations, *F*_*c*_, *F*_*e*_ and *F*_*t*_ form a feature group, i.e., energy-related features.

### Structure-related features

Generally, the edge, corner and texture are important for the structure of an image. Based on the structure tensor salient detection operator from reference [39], gradient information can be detected efficiently, and the result obtained is denoted by ***S***_T_. In addition, to strengthen the gradient feature map obtained by structure tensor salient detection, the neighbor energy is calculated, denoted as ***N***_E_, and it can be defined as

$$N_{\text{E}}(x,y)=\sum_{a=-v}^{v}\sum_{b=-v}^{v}I_{\text{S}}(x+a,y+b)$$ (13)

where (x, y) denotes the central position of ***I***_S_, *v* controls the size of the window, and (2*v*+1)×(2*v*+1) is the size of the neighborhood.

Then, the enhanced gradient feature map ***S***_NE_ is calculated as

$$S_{\text{NE}}\text{(x,y)=}S_{\text{T}}\text{(x,y)}\cdot N_{\text{E}}\text{(x,y)}$$ (14)

**Fig 3:**
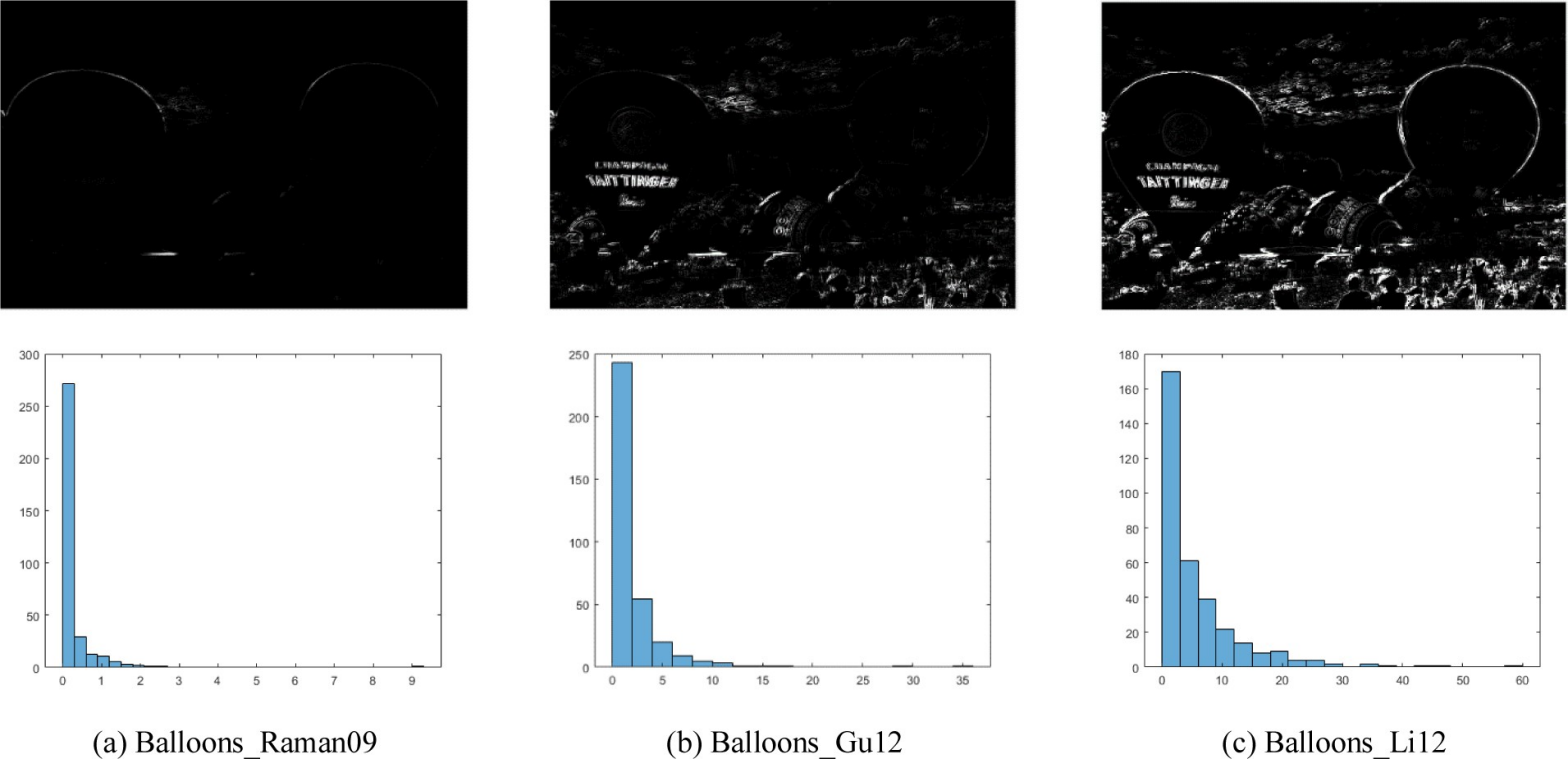
The examples of *S*_NE_ maps and distribution maps of their corresponding SVD results, where the corresponding MEF images are listed in the first line of Fig 2. The first line represents *S*_NE_ maps, the second line represents distribution maps. (a) Balloons_Raman09, (b) Balloons_Gu12, (c) Balloons_Li12.

Fig 3 gives examples of ***S***_NE_ obtained from three different MEF images corresponding to the first line of Fig 2. It can be found that Fig 3 increases the difference of structure layers among three MEF images, which is crucial for the discrimination of different distortions.

To quantitate such differences, singular value decomposition (SVD) is used to calculate the primary information of ***S***_NE_, denoted as $S_{\text{svd}}\;(t):\;t\in (1,\;2,\;...,\;q)$, where *q* is the number of the SVD diagonal coefficients. The distribution of ***S***_svd_ is enumerated in the second line of Fig 3. It can be found that different *S*_svd_ values show different and regular distributions, which can be captured by the Weibull distribution model. It can be defined as

$$f(S_{\text{svd}})=\frac{\chi }{\varepsilon }{\left(\frac{S_{\text{svd}}}{\varepsilon }\right)}^{\chi -1}\exp\left[-{\left(\frac{S_{\text{svd}}}{\varepsilon }\right)}^{\chi }\right]\;S_{\text{svd}}>0$$ (15)

where *χ* and *ε* denote the shape parameter and scale parameter.

The parameters constitute the first group of structure-related features, denoted as *F*_*w*_.

To capture the statistical differences more comprehensively, the moment features including variance, kurtosis and skewness are extracted to assist the statistical distribution properties. The obtained features are denoted by *F*_*m*_ together and regarded as the second group of structure-related features.

Except for the statistical differences’ calculation, the Local Binary Pattern (LBP) operator of local rotation invariant uniform is used on the ***S***_NE_ map to code the texture information, which can be expressed by

$$SLBP_{D,R}^{riu2}=\left\{\begin{array}{l} \sum_{o=0}^{D-1}\rho \left(S_{NE}(o)-S_{NE}(c)\right),\;\varsigma (SLBP_{D,R}^{riu2})\leq 2\\ D+1,\;otherwise \end{array}\right.$$ (16)

where *D* and *R* are the numbers of neighboring pixels and the radius, respectively. *o* and *c* stand for the center position and its neighborhood positions, respectively. *riu*2 means rotation invariant uniform pattern. *ς*(.) is the function of consistency measure calculated by the number of bit leaps, defined as

$$\begin{array}{l} \varsigma (SLBP_{D,R}^{riu2})=\left\|\rho \left(S_{NE}(D-1)-S_{NE}(c)\right)-\rho \left(S_{NE}(0)-S_{NE}(c)\right)\right\|\\ \;+\sum_{o=0}^{D-1}\left\|\rho \left(S_{NE}(o)-S_{NE}(c)\right)-\rho \left(S_{NE}(o-1)-S_{NE}(c)\right)\right\| \end{array}$$ (17)

where *ρ*(.) is the threshold function, and it can be calculated by

$$\rho \left(S_{NE}(o)-S_{NE}(c)\right)=\left\{\begin{array}{l} 1,\;S_{NE}(o)-S_{NE}(c)\geq 0\\ 0,\;otherwise\; \end{array}\right.$$ (18)

Afterward, the histogram of $SLBP_{D,R}^{riu2}$ can be calculated as

$$h(u)=\sum_{z=1}^{Z}f(SLBP_{D,R}^{riu2}(z),u)$$ (19)

$$f(x,y)=\left\{\begin{array}{l} 1,\;if\;x=y\\ 0,\;otherwise \end{array}\right.$$ (20)

where *Z* is the total number of pixels in the $SLBP_{D,R}^{riu2}$, *u* is the possible LBP pattern.

The histogram features are denoted as *F*_h_ together and regarded as the third group of structure-related features.

### Color-related features

As described in section 3.1, color information is very important for a high-quality MEF image. Since the RGB color space is highly correlated with each other, it is not proper for feature extraction. Using unrelated color space for feature extraction can eliminate the redundancy between different features, so as to perceive visual stimuli more effectively. Therefore, the RGB color space is converted into YUV color space. The obtained Y, U, and V color channels are utilized for the respective feature extraction.

For the Y color channel, the mean, standard deviation, and skewness are calculated as the first, second, and third-order moment statistics. As a result, the first group of color-related features is formed, denoted by *F*_*o*_.

Besides the *F*_*o*_, the natural scene statistic model is also used to extract features in the *U* and *V* color channels for representing the variation of color information. Specifically, *U* and *V* channel maps are processed by the local mean subtraction and divisive normalization, and then the mean subtracted contrast normalized (MSCN) coefficients are calculated, which can be given by

$$\hat{\kappa }(i,j)=\frac{\kappa (i,j)-\mu (i,j)}{\delta (i,j)+1}$$ (21)

where *k* ∈ {*U*,*V*}, $\hat{\kappa }(i,j)$ are the MSCN coefficients of *k* at the position of (x, y). *μ*(*i*, *j*) and *δ*(*i*, *j*) represent the local mean and standard deviation of *k*(*i*, *j*), respectively.

The color information of different MEF images will have different MSCN coefficients distributions, which can be caught by the generalized Gaussian distribution (GGD). The GGD can be expressed by

$$f(\omega ,\alpha ,\lambda^{2})=\frac{\alpha }{2\beta \Gamma (1/\alpha )}\cdot e^{-{\left(|\omega |/\beta \right)}^{\alpha }}$$ (22)

where $\beta =\lambda \sqrt{\Gamma (1/\alpha )\Gamma (3/\alpha )}$, *α* and *λ*^2^ are the shape and variance of the Gaussian distribution, respectively, $\Gamma \left(\cdot \right)$ is the Gamma function.

Finally, the *α* and *λ*^2^ of *U* and *V* color channel maps constitute the second group of color-related features, denoted by *F*_*n*_.

### Quality regression

With the extracted energy-related features *F*_*c*_, *F*_*e*_ and *F*_*t*_, structure-related features *F*_*w*_, *F*_*m*_ and *F*_*h*_, and color-related features *F*_*o*_, *F*_*n*_, the whole feature vector can be denoted by *F*_*all*_ *=* {*F*_*c*_, *F*_*e*_, *F*_*t*_, *F*_*w*_, *F*_*m*_, *F*_*h*_, *F*_*o*_, *F*_*n*_}. Then, random forest (RF) is adopted to learn the mapping relationship from the whole feature vector *F*_*all*_ to the subjective rating. The RF model can be expressed by

$$Q=\eta_{\text{RF}}\left(F_{all}\right)$$ (23)

where $\eta_{\text{RF}}\left(\cdot \right)$ is the mapping function by RF, and *Q* is the predicted quality score by the trained RF model.

## Experimental results

In this section, the proposed method is compared with the state-of-the-art IQA methods on the MEF image dataset. In addition, the performances of different features are tested. Then, feature selection is also conducted.

### Experimental settings

1) Dataset: The proposed method is tested on an open MEF image dataset [40], the details of the dataset are shown in Table 1. The dataset is consisting of 136 MEF images, which were obtained from the MEF algorithms, i.e., local energy weighted linear combination, global energy weighted linear combination, Li12 [9], Raman09 [8], ShutaoLi12 [11], Mertens07 [7], Gu12 [10], ShutaoLi13 [12].

**Table 1:** The details of the MEF image database [40].

No.	Source Sequences	Size	Image Source
1	Balloons	339×512×9	Erik Reinhard
2	Belgium house	512×384×9	Dani Lischinski
3	Lamp1	512×384×15	Martin Cadik
4	Candle	512×364×10	HDR Projects
5	Cave	512×384×4	Bartlomiej Okonek
6	Chinese garden	512×340×3	Bartlomiej Okonek
7	Farmhouse	512×341×3	HDR Projects
8	House	512×340×4	Tom Mertens
9	Kluki	512×341×3	Bartlomiej Okonek
10	Lamp2	512×342×6	HDR Projects
11	Landscape	512×341×3	HDRsoft
12	Lighthouse	512×340×3	HDRsoft
13	Madison capitol	512×384×30	Chaman Singh Verma
14	Memorial	341×512×16	Paul Debevec
15	Office	512×340×6	Matlab
16	Tower	341×512×3	Jacques Joffre
17	Venice	512×341×3	HDRsoft

2) Performance criteria: To validate the performance of the proposed method, three common indicators are used including the Person linear correlation coefficient (PLCC), Spearman rank order correlation coefficient (SROCC) and Root mean squared error (RMSE). For these indicators, PLCC is utilized for prediction accuracy evaluation, SROCC calculates the prediction monotonicity, and RMSE represents the prediction error. For an excellent IQA method, the PLCC and SROCC are close to 1, and RMSE is close to 0. They can be represented as

$$PLCC=\frac{\sum_{i=1}^{K}\left(Q_{i}-\bar{Q}\right)\left(M_{i}-\bar{M}\right)}{\sqrt{\sum_{i=1}^{K}{\left(Q_{i}-\bar{Q}\right)}^{2}}\sqrt{\sum_{i=1}^{K}{\left(M_{i}-\bar{M}\right)}^{2}}}$$ (24)

$$SROCC=1-\frac{6\times \sum_{i=1}^{K}l_{i}^{2}}{K\times \left(K^{2}-1\right)}$$ (25)

$$RMSE=\sqrt{\frac{1}{K}\sum_{i=1}^{K}{\left(M_{i}-Q_{i}\right)}^{2}}$$ (26)

where *Q*_*i*_ and *M*_*i*_ are the predicted quality score and the MOS value of the *i*-th MEF image, while $\bar{Q}$ and $\bar{M}$ are the mean values of *Q*_*i*_ and *M*_*i*_, respectively. *l*_*i*_ represents the rank difference of *i*-th MEF image between the objective and subjective quality assessments. *K* represents the number of MEF images.

3) Evaluation protocol: In the experiments, *k*-fold cross-validation is employed to test the performance of the proposed method. Since the MEF dataset can be divided into 17 subsets based on the number of the source MEF images, *k* is 17, 16 subsets are used for quality prediction model training, and the remaining subset is used to test. Finally, the mean performance is reported through the 17-cross verification.

### Overall performance comparison

To validate the performance of the proposed method, comparative experiments are implemented between the proposed method and 13 NR-IQA methods for ordinary and tone-mapped images on the above-mentioned MEF dataset. Among these compared methods, 10 of which are designed for ordinary images, namely DIIVINE [16], BLIND-II [17], BRISQUE [18], CurveletQA [19], GradLog [21], ConstrastQA [20], GLBP [22], OG [23], NIQMC [25], and SCORER [26]. The remaining 3 methods are designed for tone-mapped images, involving BTMQI [31], HIGRADE-1 [32], HIGRADE-2 [32]. The PLCC, SROCC and RMSE results of such comparative methods and the proposed method are shown in Table 2. Note that the best performance values are highlighted in bold.

**Table 2:** The overall performance comparison results.

Metrics	PLCC	SROCC	RMSE
DIIVINE	0.491	0.403	1.452
BLINDS-II	0.534	0.346	1.409
BRISQUE	0.414	0.380	1.517
CurveletQA	0.371	0.337	1.548
GradLog	0.631	0.567	1.293
ContrastQA	0.458	0.412	1.482
GWH-GLBP	0.163	0.113	1.645
OG	0.523	0.525	1.421
NIQMC	0.519	0.404	1.425
SCORER	0.481	0.494	1.461
BTMQI	0.452	0.343	1.487
HIGRADE-1	0.561	0.566	1.380
HIGRADE-2	0.585	0.583	1.352
Proposed	**0.701**	**0.687**	**1.191**

From Table 2, it can be drawn that, the methods designed for ordinary images show poor performance. Since they mainly describe the distortion representation of ordinary images, the features extracted by these methods cannot match the special distortion of MEF images. It is obvious that they are not proper to predict the quality of MEF images. Among them, the GradLog method exhibits relatively better performance, which may attribute to the consideration of structure information based on the gradient and Gaussian response. It can well represent the structure loss in the process of MEF, and the PLCC and SROCC values achieve the level of 0.631 and 0.567, respectively. In addition, the performance of the methods designed for tone-mapped images reaches higher PLCC and SROCC values than that of the methods designed for ordinary images. The main reason is that the MEF images and tone-mapped images both have the problem of underexposure and overexposure. Finally, the performance of our proposed method is superior to other methods analyzed above. The proposed method makes use of the idea of energy layer and structure layer decomposed from MEF image based on the joint bilateral filter to percept the detail and structure information and so on. Besides, color information is also considered. In summary, the energy-related, structure-related, and color-related features can supplement each other.

**Fig 4:**
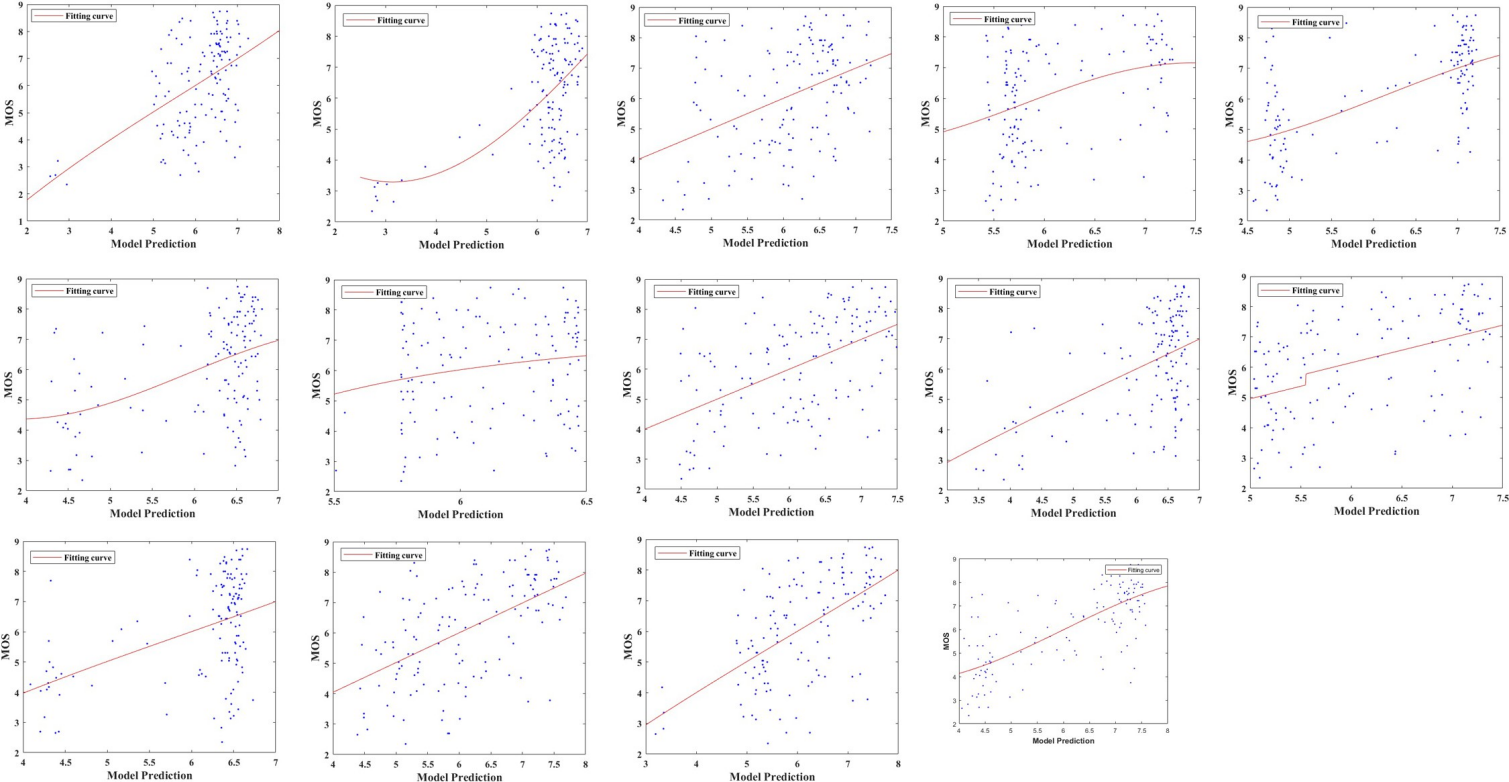
Scatter plots for the objective predicted scores and MOS values of sixteen NR-IQA methods. (a) DIIVINE [16]; (b) BLINDS_II [17]; (c) BRISQUE [18]; (d) CurveletQA [19]; (e) GradLog [21]; (f) ContrastQA [20]; (g) GLBP [22]; (h) OG [23]; (i) NIQMC [25]; (j) SCORER [26]; (k) BTMQI [31]; (l) HIGRADE-1 [32]; (m) HIGRADE-2 [32]; (n) Proposed.

To illustrate the performance comparison results more intuitively, Fig 4 gives out the scatter plots of the proposed method as well as the competing methods. As can be found, the proposed method has superior convergence than the other comparative methods and the points can be fitted by the logistic function better. So, it is obvious that the proposed method achieves better performance than other comparative methods.

### Ablation study

Through the above overall performance comparison, the proposed method has been validated to have superior performance to other methods for ordinary and tone-mapped images. However, different features constitute the whole feature vector, and their own contribution should be tested. The results are given out in Table 3, including the individual features and the combinations of different features. Among the performances of different features, it can be drawn that, the energy-related features, the structure-related features and the color-related features contribute equally, and the combination of them maximizes performance.

**Table 3:** Performance comparison of different feature combinations.

Features	PLCC	SROCC	RMSE
*F_c_*	0.356	0.324	1.558
*F_e_*	0.368	0.338	1.550
*F_t_*	0.453	0.348	1.486
*F_c_*, *F_e_*, *F_t_*	**0.556**	**0.510**	**1.385**
*F_w_*	0.362	0.326	1.553
*F_m_*	0.386	0.311	1.542
*F_h_*	0.401	0.319	1.527
*F_w_*, *F_m_*, *F_h_*	**0.522**	**0.454**	**1.422**
*F_o_*	0.475	0.451	1.466
*F_n_*	0.505	0.510	1.439
*F_o_*, *F_n_*	**0.514**	**0.457**	**1.430**
all	**0.701**	**0.687**	**1.191**

Even so, the combination of multiple classes of features may cause redundancy. Therefore, feature selection is utilized based on the RF to determine the final feature vector, and the results are listed in Table 4. It can be clearly found that the 15-dimension feature vector selected from the whole extracted features obtained the best performance.

**Table 4:** Performance comparison with features of different dimensions.

Feature dimensions	PLCC	SROCC	RMSE
1–10	0.714	0.693	1.167
1–15	**0.742**	**0.710**	**1.118**
1–31	0.701	0.687	1.191

## Discussion

This paper presents a MEF-IQA method that can blindly predict the quality of MEF images. Through the above experiments, it can be demonstrated that the proposed method has a better performance, competing with several other IQA methods. Its good performance depends on the consideration of the detail, structure, and color distortions, which significantly affect the quality of MEF images. However, further consideration of knowledge in the field of MEF algorithms is also meaningful for the MEF-IQA, i.e., the cause of distortion formation can be considered while mining the distortion characteristics. In fact, although the proposed method has good performance, there is still room for improvement. The MEF-IQA method can be further improved by incorporating such knowledge. Besides, it can be considered to combine high-level semantic features to enhance the ability of feature representation.

## Conclusion

In this paper, a novel blind quality assessment method considering the detail, structure and color characteristics is proposed for the MEF images. Inspired by the image fusion process, the MEF image is decomposed into two components (i.e., the energy layer and structure layer) for representing the detail and structure information through the joint bilateral filtering firstly. Then, corresponding features are extracted from them to describe the detail and structure distortion characteristics of MEF images. Besides, color space conversion is also utilized for color-related features extraction to perceive color degradation. Finally, to achieve the best performance, feature selection is conducted based on the random forest. Experimental results demonstrate the superiority of the proposed method.

## References

[1] Li H, Chan TN, Qi X, Xie W. Detail-preserving multi-exposure fusion with edge-preserving structural patch decomposition. IEEE Trans. Circuits Syst. Video Technol. 2021; 31(11): 4293–4304.

[2] Sun C, Song K, Su J, Yan Y, Zhang T. A Multi-Exposure Fusion Method for Reflection Suppression of Curved Workpieces. IEEE Trans. Instrum. Meas. 2021; 71: 5021104.

[3] Qi Y, Zhou S, Zhang Z, Luo S, Lin X, Wang L, et al. Deep unsupervised learning based on color un-referenced loss functions for multi-exposure image fusion. Inf. Fusion. 2021; 66: 18–39.

[4] Shen L, Chen X, Pan Z, Fan K, Li F, Lei J. No-reference stereoscopic image quality assessment based on global and local content characteristics. Neurocomputing. 2021; 424: 132–142.

[5] Burt P, Kolczynski R. Enhanced image capture through fusion. In proceedings of 4th Int. Conf. Comput. Vis. 1993; 173–182.

[6] Goshtasby A. Fusion of multi-exposure images. Image Vis. Comput. 2005; 23(6): 611–618.

[7] Mertens T, Kautz J, Van Reeth F. Exposure fusion: A simple and practical alternative to high dynamic range photography. Comput. Graph. Forum. 2009; 28(1): 161–171.

[8] Raman S, Chaudhuri S. Bilateral filter based compositing for variable exposure photography. In Proceedings of the Eurographics (Short Papers), Munich, Germany, 30 March-3 April 2009; 1–3.

[9] Li Z, Zheng J, Rahardja S. Detail-enhanced exposure fusion. IEEE Trans. Image Process. 2012; 21(11): 4672–4676. doi: 10.1109/TIP.2012.2207396 22801512

[10] Gu B, Li W, Wong J, Zhu M, Wang M. Gradient field multi-exposure images fusion for high dynamic range image visualization. J. Vis. Commun. Image Represent. 2012; 23(4): 604–610.

[11] Li S, Kang X. Fast multi-exposure image fusion with median filter and recursive filter. IEEE Trans. Consum. Electron. 2012; 58(2): 626–632.

[12] Li S, Kang X, Hu J. Image fusion with guided filtering. IEEE Trans. Image Process. 2013; 22(7): 2864–2875. doi: 10.1109/TIP.2013.2244222 23372084

[13] Ma K, Duanmu Z, Yeganeh H, Wang Z. Multi-exposure image fusion by optimizing a structural similarity index. IEEE Trans. Comput. Imag. 2017; 4(1): 60–72.

[14] Ma K, Duanmu Z, Zhu H, Fang Y, Wang Z. Deep guided learning for fast multi-exposure image fusion. IEEE Trans. Image Process. 2019; 29: 2808–2819. doi: 10.1109/TIP.2019.2952716 31751238

[15] Yu G, Hou C, Yan W, Choi L, Zhou T, Hou Y. Blind quality assessment for screen content images via convolutional neural network. Digital Signal Process. 2019; 91: 21–30.

[16] Moorthy AK, Bovik AC. Blind image quality assessment: From natural scene statistics to perceptual quality. IEEE Trans. Image Process. 2011; 20(12): 3350–3364. doi: 10.1109/TIP.2011.2147325 21521667

[17] Saad MA, Bovik AC, Charrier C. Blind image quality assessment: A natural scene statistics approach in the DCT domain. IEEE Trans. Image Process. 2012; 21(8): 3339–3352. doi: 10.1109/TIP.2012.2191563 22453635

[18] Mittal A, Moorthy AK, Bovik AC. No-reference image quality assessment in the spatial domain. IEEE Trans. Image Process. 2012; 21(12): 4695–4708. doi: 10.1109/TIP.2012.2214050 22910118

[19] Liu L, Dong H, Huang H, Bovik AC. No-reference image quality assessment in curvelet domain. Signal Process. Image Commun. 2014; 29(4): 494–505.

[20] Fang Y, Ma K, Wang Z, Lin W, Fang Z, Zhai G. No-reference quality assessment of contrast-distorted images based on natural scene statistics. IEEE Signal. Process. Lett. 2014; 22(7): 838–842.

[21] Xue W, Mou X, Zhang L, Bovik AC, Feng X. Blind image quality assessment using joint statistics of gradient magnitude and Laplacian features. IEEE Trans. Image Process. 2014; 23(11): 4850–4862. doi: 10.1109/TIP.2014.2355716 25216482

[22] Li Q, Lin W, Fang Y. No-reference quality assessment for multiply-distorted images in gradient domain. IEEE Signal Process. Lett. 2016; 23(4): 541–545.

[23] Liu L, Hua Y, Zhao Q, Huang H, Bovik AC. Blind image quality assessment by relative gradient statistics and adaboosting neural network. Signal Process Image Commun. 2016; 40: 1–15.

[24] Gu K, Lin W, Zhai G, Yang X, Zhang W, Chen CW. No-reference quality metric of contrast-distorted images based on information maximization. IEEE Trans. Cybern. 2016; 47(12): 4559–4565. doi: 10.1109/TCYB.2016.2575544 27323391

[25] Jiang Q, Peng Z, Yue G, Li H, Shao F. No-reference image contrast evaluation by generating bidirectional pseudoreferences. IEEE Trans. Industrial Informatics. 2020; 17(9): 6062–6072.

[26] Oszust M. Local feature descriptor and derivative filters for blind image quality assessment. IEEE Signal Process. Lett. 2019; 26(2): 322–326.

[27] Ma K, Zeng K, Wang Z. Perceptual quality assessment for multi-exposure image fusion. IEEE Trans. Image Process. 2015; 24(11): 3345–3356. doi: 10.1109/TIP.2015.2442920 26068317

[28] Xing L, Cai L, Zeng H, Chen J, Zhu J, Hou J. A multi-scale contrast-based image quality assessment model for multi-exposure image fusion. Signal Process. 2018; 145: 233–240.

[29] Martinez J, Pistonesi S, Maciel M, Flesia A. Multi-scale fidelity measure for image fusion quality assessment. Inf. Fusion. 2019; 50: 197–211.

[30] Xu J, Zhou W, Li H, Li F, Jiang Q. Quality assessment of multi-exposure image fusion by synthesizing local and global intermediate references. Displays. 2022; 74: 102188.

[31] Gu K, Wang S, Zhai G, Ma S, Yang X, Lin W, et al. Blind quality assessment of tone-mapped images via analysis of information, naturalness, and structure. IEEE Trans. Multimed. 2016; 18(3): 432–443.

[32] Kundu D, Ghadiyaram D, Bovik AC, Evans BL. No-reference quality assessment of tone-mapped HDR pictures. IEEE Trans. Image Process. 2017; 26(6): 2957–2971. doi: 10.1109/TIP.2017.2685941 28333633

[33] Yue G, Hou C, Gu K, Mao S, Zhang W. Biologically inspired blind quality assessment of tone-mapped images. IEEE Trans. Industrial Electron. 2017; 65(3): 2525–2536.

[34] Yue G, Hou C, Zhou T. Blind quality assessment of tone-mapped images considering colorfulness, naturalness, and structure. IEEE Trans. Industrial Electron. 2018; 66(5): 3784–3793.

[35] Jiang Q, Shao F, Lin W, Jiang G. BLIQUE-TMI: Blind quality evaluator for tone-mapped images based on local and global feature analyses. IEEE Trans. Circuits Syst. Video Technol. 2017; 29(2): 323–335.

[36] Wang X, Jiang Q, Shao F, Gu K, Zhai G, Yang X. Exploiting local degradation characteristics and global statistical properties for blind quality assessment of tone-mapped HDR images. IEEE Trans. Multimed. 2020; 23: 692–705.

[37] Tomasi C, Manduchi R. Bilateral filtering for gray and color images. IEEE International Conf. Computer Vision, Bombay, India, 1998; 893–846.

[38] Ferzli R, Lina J. A no-reference objective image sharpness metric based on the notion of just noticeable blur (JNB). IEEE Trans. Image Process. 2009; 18(4): 717–728. doi: 10.1109/TIP.2008.2011760 19278916

[39] Zhou Z, Li S, Wang B. Multi-scale weighted gradient-based fusion for multi-focus images. Inf. Fusion. 2014; 20: 60–72.

[40] Multi-exposure Fusion Image Database. Available from: https://ivc.uwaterloo.ca/database/M-EF.html (accessed on 11 July 2015).

